# Appointment

**Published:** 1852-09

**Authors:** 


*Appointment.*—We learn that Dr. Robert E. Rogers, of the University of Virginia, has been appointed professor of chemistry in the University of Pennsylvania, in place of his brother, the late Dr. James B. Rogers. This is a first rate appointment, for we do not know a man who *teaches* chemistry better than Dr. R., in the whole country.—*Stethoscope.*

